# Case Report: Complete Response to Antiangiogenesis and Immune Checkpoint Blockade in an Unresectable MMR-Deficient Leiomyosarcoma Harboring Biallelic Loss of PTEN

**DOI:** 10.3389/fonc.2022.802074

**Published:** 2022-02-14

**Authors:** Xi Guo, Suyao Li, Hanxing Tong, Yong Zhang, Yuan Ji, Rongyuan Zhuang, Chenlu Zhang, Yang You, Weiqi Lu, Yuhong Zhou

**Affiliations:** ^1^ Department of Medical Oncology, Zhongshan Hospital, Fudan University, Shanghai, China; ^2^ Department of Clinic, Cancer Center, Zhongshan Hospital, Fudan University, Shanghai, China; ^3^ Department of General Surgery, Zhongshan Hospital, Fudan University, Shanghai, China; ^4^ Department of Pathology, Zhongshan Hospital, Fudan University, Shanghai, China

**Keywords:** leiomyosarcoma, PTEN, antiangiogenesis, immune checkpoint blockade, immunotherapy combined therapy

## Abstract

**Background:**

Leiomyosarcoma (LMS) is a malignant smooth muscle neoplasm, in which the efficacy of immune checkpoint blockade (ICB) is very limited. What is worse, loss of *PTEN*, known as a negative factor for ICB, frequently occurred in LMS. Seeking new strategies for LMS patients harboring loss of *PTEN* is important and challenging.

**Case Presentation:**

A 42-year-old Chinese male was diagnosed as having unresectable LMS of the iliopsoas. After the failure of two prior chemotherapy regimens, whole-exome sequencing revealed that tumor tissue had high tumor mutation burden (689 Muts), high microsatellite instability, and some somatic mutations, including *PTEN* (copy number loss and p.N323fs), *MSH6* (p.F1088fs), *TP53* p.R273C, *ASXL1* p.G645fs, *ATR* p.S1843P, and *CDKN2A* p.A118P. Then, antiangiogenic agent (pazopanib or anlotinib) plus pembrolizumab was administered from January 2 to August 6, 2018. However, pazopanib was stopped on June 18 due to the grade 2/3 adverse effect of hand–foot skin reaction, and anlotinib was administered. Considering that the tumor shrunk after immunotherapy, he underwent radical resection on September 6, 2018. The final pathological diagnosis confirmed pathologic complete response (CR). Until the latest follow-up (September 15, 2021), no progressive disease was observed and total disease-free survival has exceeded 36 months.

**Conclusion:**

We presented a patient with an unresectable mismatch repair (MMR)-deficient LMS harboring biallelic loss of PTEN who achieved CR from a combination strategy of antiangiogenesis plus pembrolizumab. Such a strategy might be a promising strategy to overcome the ICB resistance caused by the loss of PTEN. Such conclusions need to be further confirmed in further investigations.

## Introduction

Leiomyosarcoma (LMS) is a malignant smooth muscle neoplasm that consists of about 15%–20% of soft tissue sarcomas (STSs) and frequently originates in sites of the uterus and retroperitoneum (1, 2). Currently, radical surgery is still the dominant curative modality for early-stage LMS. For unresectable or advanced LMS, chemotherapy is regarded as a standard strategy. Besides, antiangiogenesis agents, such as pazopanib and anlotinib, have also been proven effective in a variety of STSs (3–6).

Until now, immune checkpoint blockade (ICBs) is becoming a promising treatment strategy for various tumors. For example, pembrolizumab has brought significant clinical benefits for patients with microsatellite instability-high and/or mismatch repair deficient (MSI-H/dMMR) solid tumors in the pan-cancer cohort (7). Although promising, only one dMMR STS was included in the aforementioned cohort, and the patient, unfortunately, developed progressive disease. In another retrospective study, seven LMS patients treated with nivolumab were included. Noted that three patients achieved stable disease (SD), and no patient achieved partial response (PR) or complete response (CR) (8). To the best of our knowledge, only a few cases reported that patients with LMS could achieve a response to ICB alone (9). What is worse, loss of PTEN, known as a negative factor of ICBs, occurs frequently in LMS (2, 10, 11). Recently, some clinical trials explored the combination strategies of ICBs and antiangiogenesis to alleviate ICB resistance. For instance, the combination strategy of axitinib and pembrolizumab was explored in 30 patients with STS, including six LMS patients. The median progression-free survival (PFS) was 5.4 months, and the best objective response rate (ORR) was 21.9% (12). Taken together, the efficacy of ICB in LMS is minimal. Seeking novel immunotherapy strategies for LMS patients harboring loss of PTEN is still important and challenging. Herein, we firstly reported an unresectable LMS patient harboring biallelic PTEN loss who achieved CR after antiangiogenesis plus ICB.

## Case Presentation

**Figure 1:**
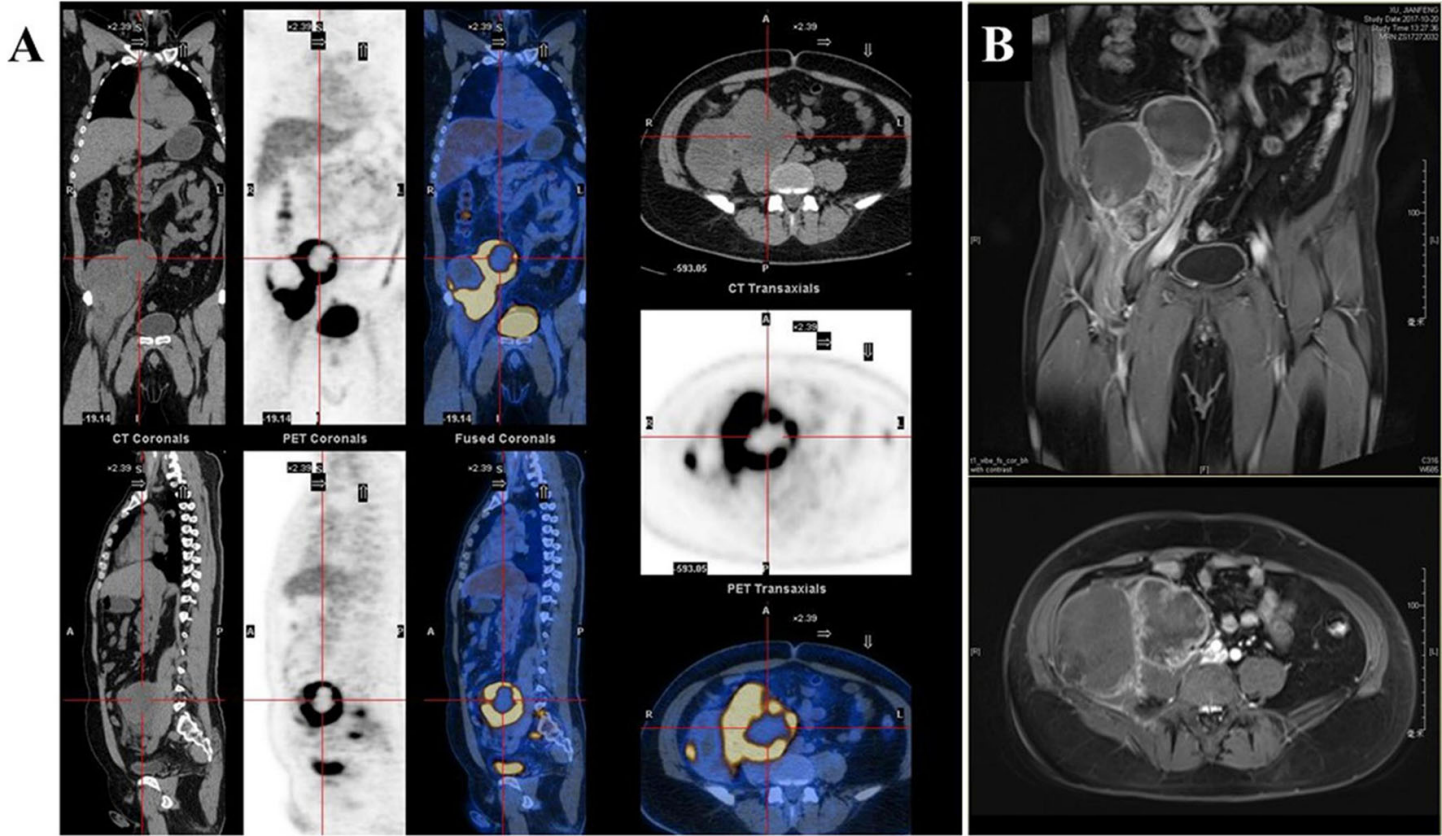
**(A)** Positron emission tomography/computed tomography (PET/CT). **(B)** Magnetic resonance imaging (MRI) demonstrated a malignant tumor involving both the right psoas major muscle and anterior iliac fossa.

**Figure 2:**
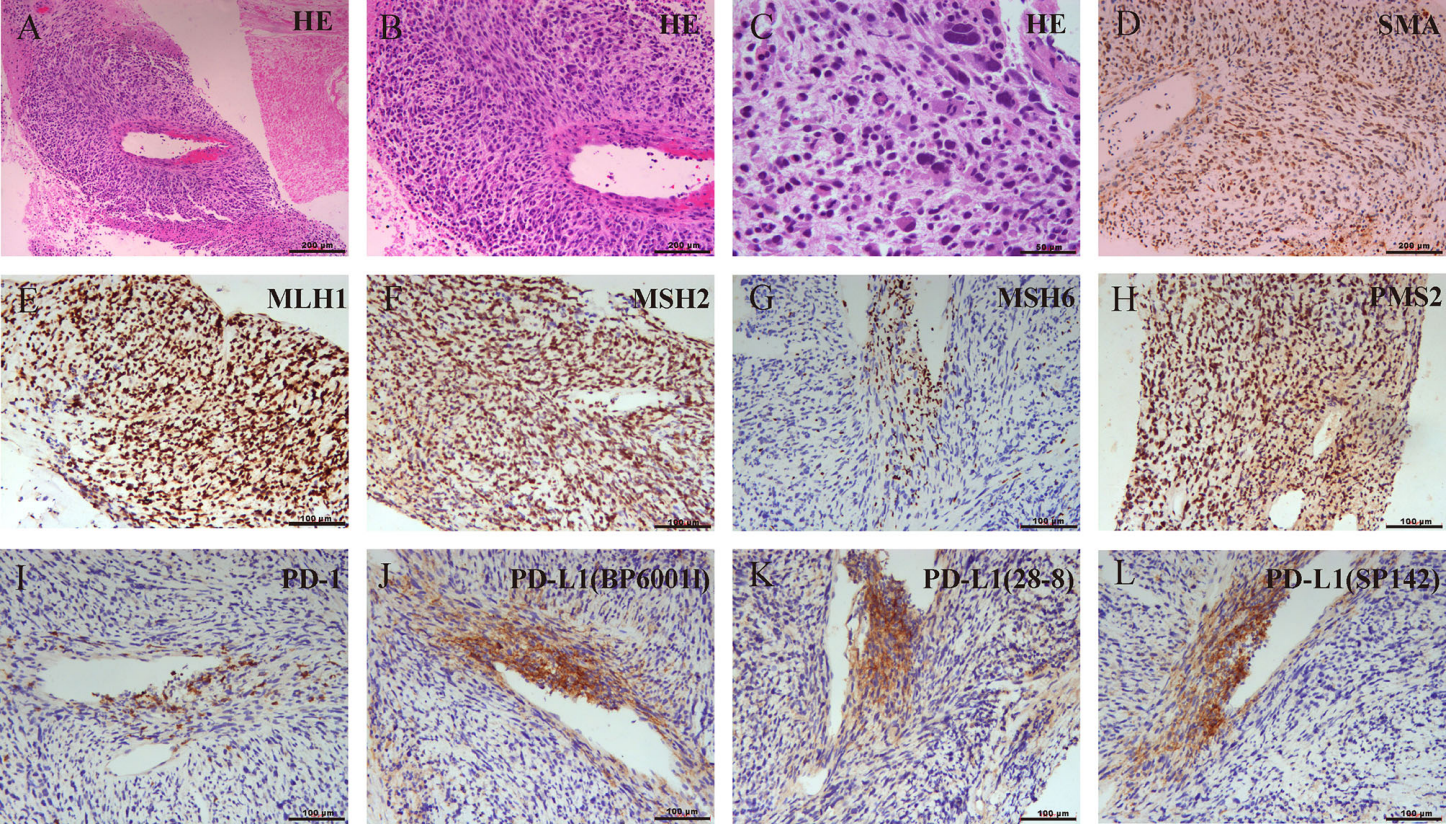
**(A–C)** Biopsy pathology confirmed mismatch repair (MMR)-deficient leiomyosarcoma (LMS) and the expression of programmed cell death protein-1/programmed cell death-ligand 1(PD-1/PD-L1) using different antibodies. The pathological lesion was measured by H&E staining. **(D)** Strong positive response of alpha-smooth muscle actin (a-SMA). **(E–H)** Absence of MSH6 staining and positive staining for MLH1, MSH2, and PMS2 indicated MSH6 deficiency. **(I)** Immunohistochemistry findings for PD-1. **(J–L)** Immunohistochemistry findings for PD-L1 using BP60001L, 28-8, or SP142.

**Figure 3:**
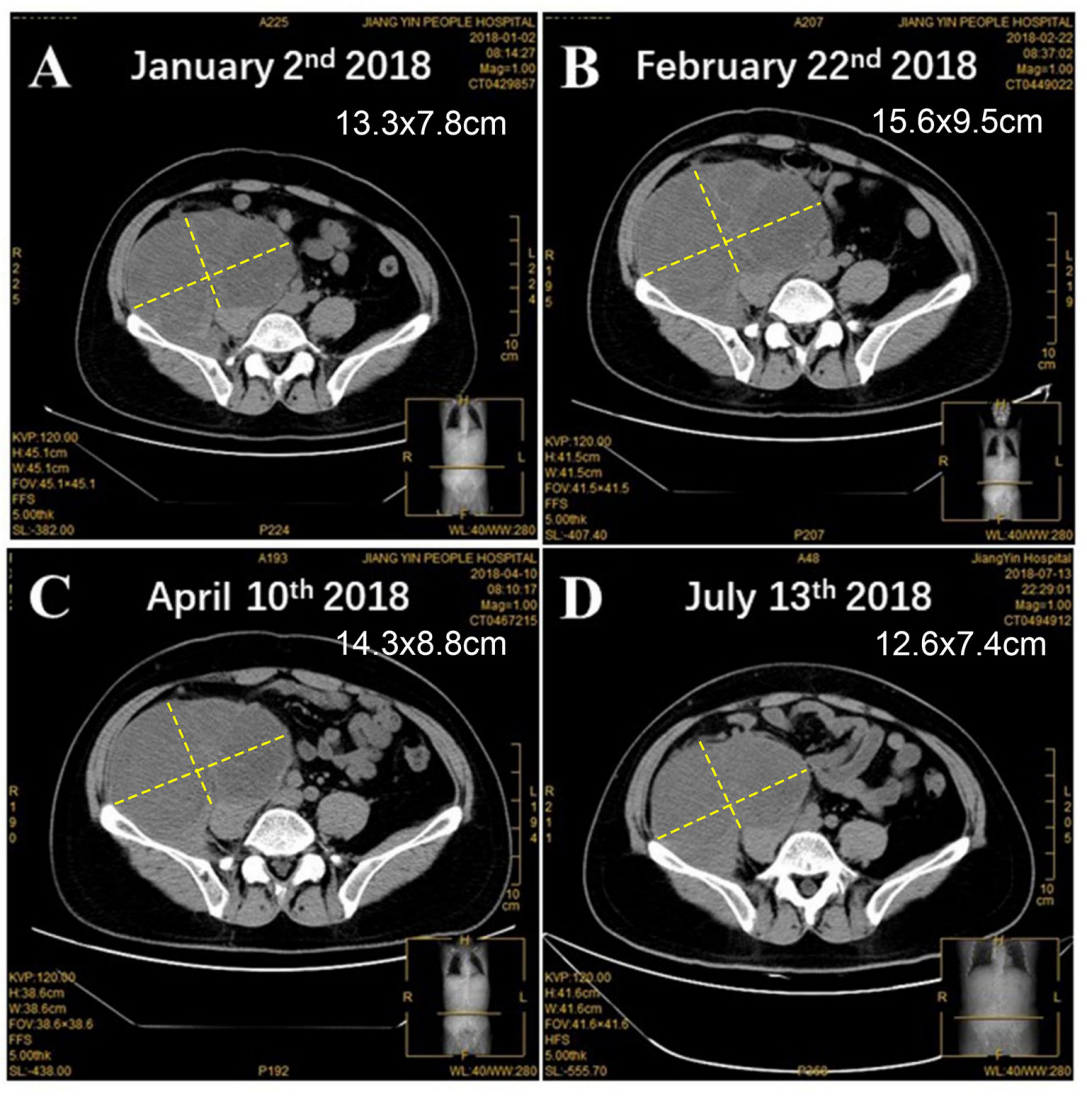
The changes of tumor lesion. **(A)** Before and after the treatment of antiangiogenesis and pembrolizumab for **(B)** 1 month, **(C)** 3 months, and **(D)** 6 months.

**Figure 4:**
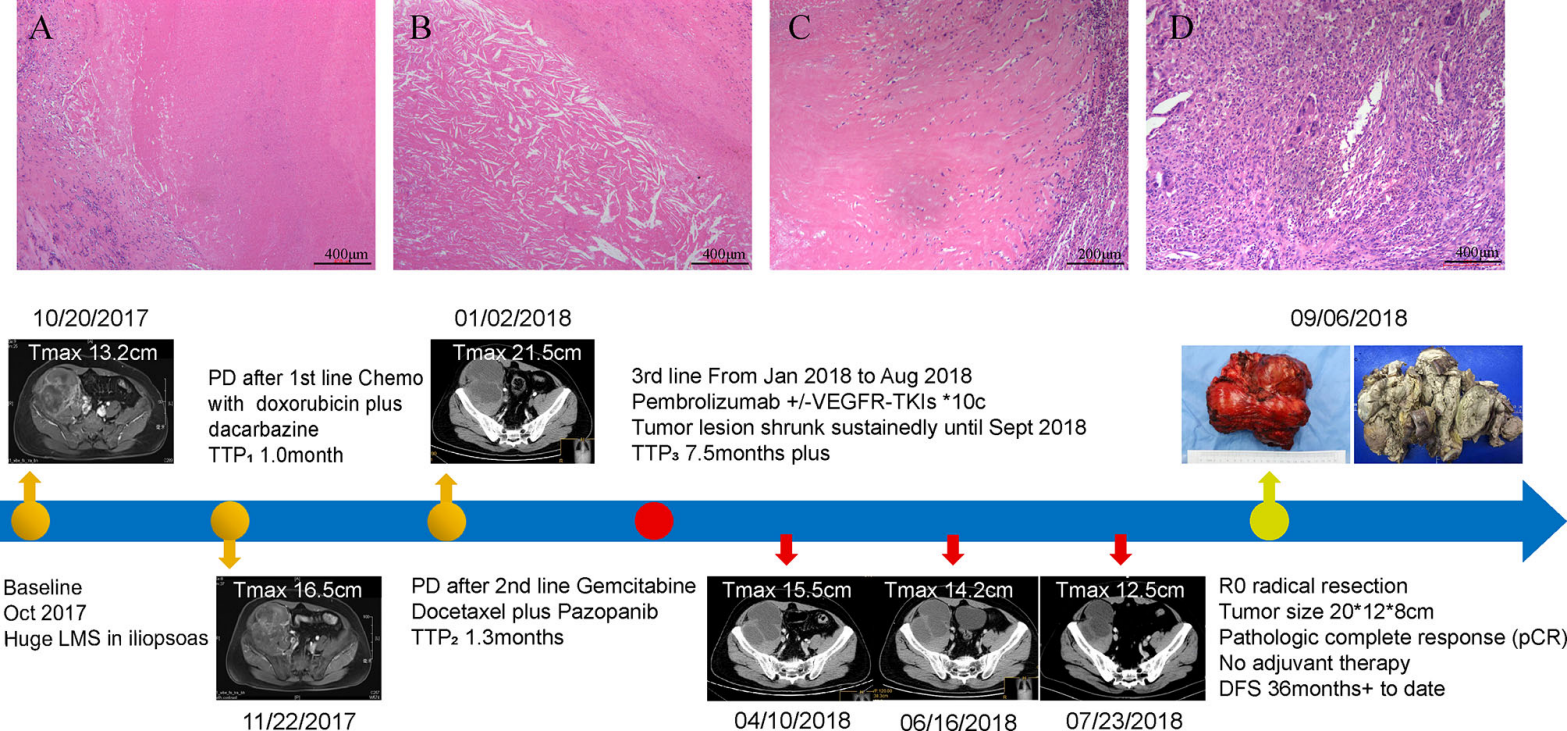
Surgical pathology confirmed a pathological complete response (pCR) in this patient and the timeline of interventions and outcomes. **(A)** A variable extent of coagulative tumoral necrosis. **(B)** Cholesterol crystals around the tumoral necrosis. **(C)** Collagen fibers around the periphery of tumoral necrosis. **(D)** Histiocytes and multinucleated giant cells in the tumor. The bottom panel shows clinical interventions and outcomes of the patient.

A 42-year-old Chinese male was admitted to Zhongshan Hospital in October 2017, who had presented right buttock numbness for 2 years. His performance status (PS) score was 1. Positron emission tomography/computed tomography (PET/CT) demonstrated a malignant tumor that involved both the right psoas major muscle and anterior iliac fossa (**Figure 1A**). The result of magnetic resonance imaging (MRI) suggested that the tumor size was 131.8 mm × 101.1 mm, which was consistent with that of PET/CT (**Figure 1B**). Finally, the patient was confirmed to have a pathologic diagnosis of cT2bNxM0, stage IIB LMS (**Figures 2A**–**D**). Considering that the huge mass had invaded the pelvic wall, surgical resection was not recommended. The patient was administered first-line chemotherapy of liposomal Adriamycin (60 mg, day 1, every 3 weeks) plus dacarbazine (750 mg, days 1–2, every 3 weeks). After failure, he was administered second-line treatment strategy of gemcitabine (1.3 g, days 1 and 8), docetaxel (130 mg, day 8, every 3 weeks), and pazopanib (400 mg, once a day) in December 2017. Unfortunately, progressive disease was still developed in January 2018. To search for potential treatment opportunities, tumor tissue DNA was subjected to a next-generation sequencing-based assay. The whole-exome sequencing (WES) results showed that the patient had a high tumor mutation burden (TMB; 689 Muts) and MSI-H. Besides, some cancer-related gene somatic mutations also occurred, including biallelic loss of PTEN (copy number loss and p. N323fs), MSH6 (F1088fs), TP53 p.R273C, ASXL1 p.G645fs, ATR p.S1843P, and CDKN2A p.A118P. The expression of protein MSH6 was significantly lower compared with those of other proteins (MLH1, MSH2, or PMS2) (**Figures 2E**–**H**). Immunohistochemical staining revealed that PD-1 expression was 2% positive in tumor cells, negative in the stroma (**Figure 2I**) and PD-L1(BP60001L, 28-8,SP142) expression was negative in tumor cells, 2% positive in the stroma (**Figures 2J–L**). After Multi-Disciplinary Treatment (MDT) discussion, the patient received antiangiogenesis (pazopanib or anlotinib) plus pembrolizumab from January 10 to August 6, 2018. **Figures 3A**–**C** showed that the tumor lesion shrunk under the treatment of pembrolizumab (200 mg every 3 weeks) plus pazopanib (400 mg once a day). However, pazopanib was stopped on June 18 due to the grade 2/3 adverse effect (AE) of hand–foot skin reaction. Subsequently, anlotinib (12 mg, days 1–14, every 3 weeks) was administered, and the tumor continued to shrink (**Figure 3D**). Other AEs during the whole treatment were grade 1 hair discoloration, on which no management was performed. The patient underwent radical resection of retroperitoneal sarcoma, repair of abdominal wall defect, and resection of small bowel mesentery on September 6, 2018. The operation site was marked with a titanium clip, and a biological patch was placed. As shown in **Figures 4A**–**D**, final pathological diagnosis results confirmed a pathologic complete response (pCR).

**Figure 5:**
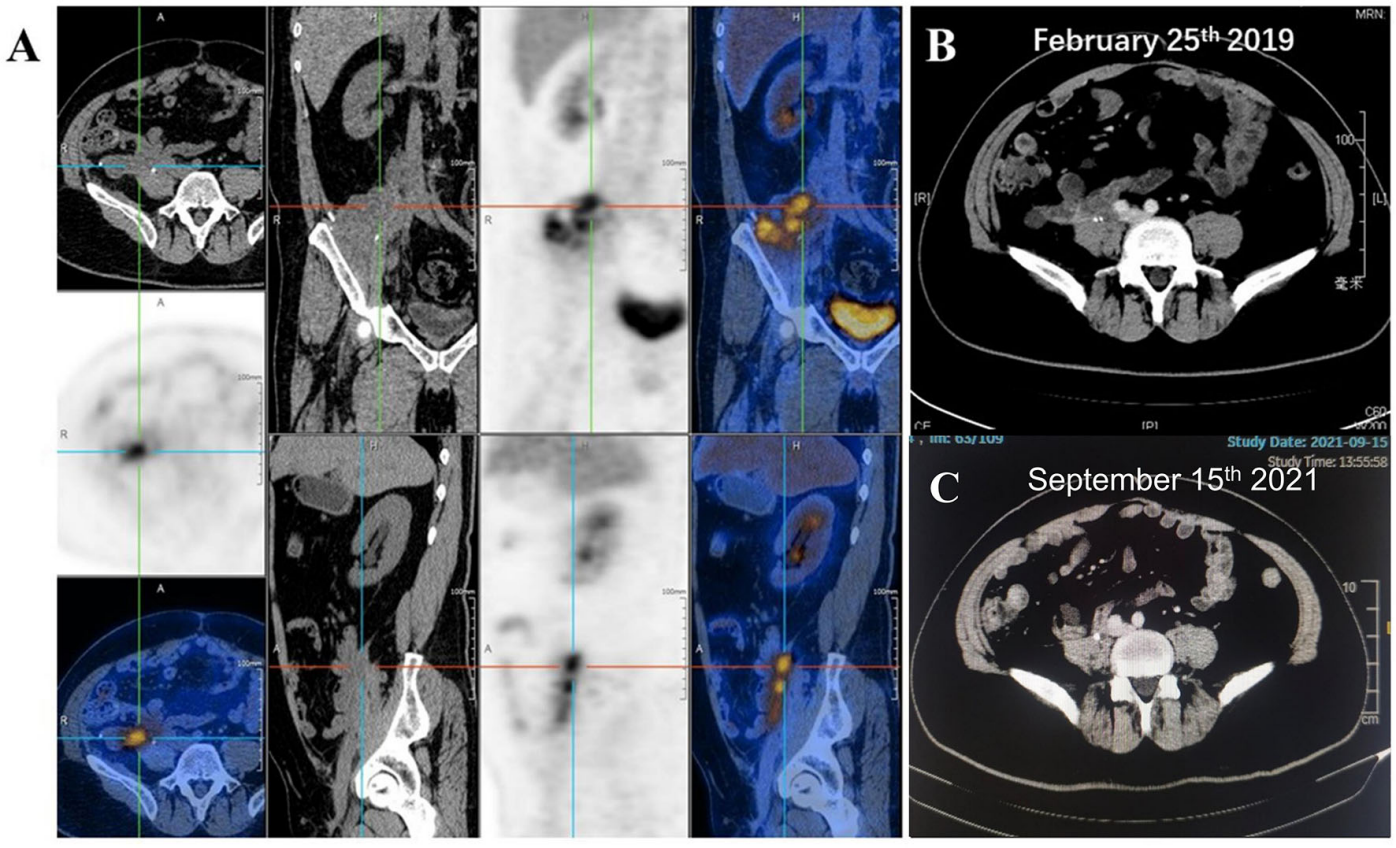
**(A)** Positron emission tomography/computed tomography (PET/CT) suggested a high local metabolic activity during the follow-up of March 2019. CT scans for the patient on **(B)** February 25 and **(C)** September 15, 2021.

During the follow-up in March 2019, PET/CT results in **Figure 5A** indicated a high local metabolic activity, which implied a suspected lesion. And the CT scan (**Figure 5B**) was consistent with the PET/CT results. However, pathologic diagnosis for lesion analysis was unrealistic due to the existence of the pelvic wall bone and the biological patch. In view of the situation above, MDT assumed that the high metabolic activity might be attributed to the inflammatory reaction caused by the biological patch and recommended the patient’s close follow-up. Such supposition was confirmed in the subsequent follow-up, and no progressive disease was observed until the latest follow-up (September 15, 2021; **Figure 5C**). Currently, the total disease-free survival (DFS) has exceeded 36 months and the follow-up is still ongoing.

## Discussion

LMS is a malignant smooth muscle neoplasm that occurs frequently in the uterus, retroperitoneum, buttock, and so on (2, 13). LMS of the iliopsoas is rare and presents unique challenges in management, since it is relatively inaccessible surgically due to being close to the ureter, aorta, inferior vena cava, spine, and the iliac vessels (14). In this work, we firstly presented an unresectable patient with LMS of the iliopsoas who achieved CR to antiangiogenesis plus ICB. The final DFS has exceeded 36 months.

Antiangiogenesis, such as pazopanib and anlotinib, has demonstrated promising efficacy for the treatment of advanced STS. Pazopanib is an orally multitargeted receptor tyrosine kinase (RTK) inhibitor, involving vascular endothelial growth factor (VEGF) receptors (VEGFRs), platelet-derived growth factor receptors (PDGFRs), and c-kit (4). In a randomized phase III clinical trial (PALETTE), significantly better PFS was observed in the patients treated with pazopanib compared with placebo (4.6 vs. 1.6 months) (5). Pazopanib has been approved by the Food and Drug Administration (FDA) for STS. Similarly, anlotinib, another orally multitargeted RTK inhibitor, was also approved for STS in China (15). In a phase II clinical trial, 166 patients with advanced STS were enrolled, including malignant fibrous histiocytoma (MFH, N = 19), liposarcoma (LPS, N = 13), LMS (N = 26), synovial sarcoma (SS, N = 47), or other sarcomas (N = 30) (16). The median PFS was 5.63 months, and the ORR was 11.45%. Note that the ORR for LMS was 7.67%. In another phase IIB clinical trial, 233 patients were included who were randomized 2:1 to receive anlotinib (N = 158) or placebo (N = 75) (6). Anlotinib treatment significantly improved the median PFS relative to the control (6.27 vs. 1.47 months, p < 0.0001). For LMS (N = 41), a similar result was observed (median PFS, 5.83 months vs. 1.43 months, p < 0.0001).

Recently, ICBs are becoming another promising treatment strategy for various tumors. However, LMS is usually regarded as a “cold tumor”, and ICBs are not applicable. It was reported that LMS was more common among patients who are immunosuppressive such as allograft transplantation receivers and HIV-infected patients (17). In SARC028, 10 LMS patients were enrolled to test the efficacy of single-agent pembrolizumab and no patient responded (18). Additionally, in Alliance A091401 study, 14 STS patients treated with nivolumab and ipilimumab were included (19). Confirmed responses were seen in patients with uterine LMS (N = 1) and non-uterine LMS (N = 1). What is worse, loss of PTEN might contribute to the ICB treatment resistance, which is frequently downregulated in LMS (2, 10, 11). Previous works suggested that loss of PTEN is usually caused by gene mutation, deletion, transcriptional silencing, or protein instability (20). Tumors with biallelic PTEN loss had significantly lower levels of mRNA expression of PDCD1, CD8A, IFNG, PRF1, and GZMA compared to PTEN-wild-type tumors (p < 0.05 for all) (11, 21).

Recently, some clinical trials explored the combination strategies of ICBs and antiangiogenesis treatment to alleviate the ICB resistance. Such synergistic effect has obtained some exciting outcomes in some tumors, such as advanced clear cell renal cell carcinoma and gastric or colorectal cancer (22–24). As for STSs, the combination strategy of axitinib and pembrolizumab was also explored in 30 STS patients, including alveolar soft part sarcoma(36%), undifferentiated pleomorphic sarcoma (15%), LMS (18%), other (30%). In the whole cohort, the median PFS was 5.4 months and the best ORR was 21.9% (12). In the present case, an unresectable LMS patient harboring biallelic loss of PTEN achieved CR to antiangiogenesis plus pembrolizumab. The dMMR/MSI-H and TMB-H may provide a potential mechanism for the successful management of the LMS. Though MMR deficiency occurs infrequently in LMS, it has been reported in some studies, and the MMR-deficient status points to some LMS patients as potentially sensitive to immunotherapy (25–27). However, in a clinical trial of pembrolizumab, one dMMR sarcoma patient enrolled still developed progressive disease (7). In KEYNOTE-158 study, which recruited 233 patients with advanced non-colorectal dMMR cancer, ORR was only 34.3% (28). So, for dMMR LMS patients who do not respond well to ICB, identifying genomic features through WES and taking genetic and epigenetic characteristics into consideration may be helpful (29). When somatic mutations like PTEN were detected, combination therapy with ICB may be more effective. Whether such combination strategy could overcome the resistance caused by the loss of PTEN is another important issue. Previous works suggested that loss of PTEN could result in unopposed Phosphatidylinositol-3 kinase (PI3K) activity and subsequently promote the VEGF expression on endothelial cells, which was regarded as a key tumor-associated factor that induces changes in blood vessel permeability and architecture (10). Hegde et al. (30) reported that anti-VEGF could contribute to reprogramming the tumor milieu from an immunosuppressive to an immune permissive microenvironment (30, 31). This case highlighted that antiangiogenesis might be an optimal combination partner for ICBs, and such a combination strategy might be a promising strategy for dMMR LMS, even for LMS harboring biallelic loss of PTEN. The exact mechanism should be explored in further exploration.

## Conclusion

Taken together, this is the first report related to an antiangiogenic agent plus pembrolizumab in an unresectable dMMR LMS harboring biallelic loss of PTEN. The combination strategy of ICB plus antiangiogenesis might be a promising strategy to overcome the resistance caused by the loss of PTEN. Such conclusions need to be further confirmed in further investigations.

## Data Availability Statement

The original contributions presented in the study are included in the article/supplementary material. Further inquiries can be directed to the corresponding authors.

## Ethics Statement

The studies involving human participants were reviewed and approved by the Ethics Committee of Zhongshan Hospital of Fudan University. The patients/participants provided their written informed consent to participate in this study.

## Author Contributions

XG, SL, HT, YZ and YJ collected the clinical information, diagnostic information, therapeutic information, and images of the patients. XG and SL wrote the article. SL and CZ identified the case and submitted the article. RZ and YY revised the article. YHZ and WL proofread the article. XG and SL have contributed equally to this work. All authors contributed to the article and approved the submitted version.

## Funding

This study was supported by the Beijing Xisike Clinical Oncology Research Foundation (No. Y-sy2018-148).

## Conflict of Interest

The authors declare that the research was conducted in the absence of any commercial or financial relationships that could be construed as a potential conflict of interest.

## Publisher’s Note

All claims expressed in this article are solely those of the authors and do not necessarily represent those of their affiliated organizations, or those of the publisher, the editors and the reviewers. Any product that may be evaluated in this article, or claim that may be made by its manufacturer, is not guaranteed or endorsed by the publisher.
